# Imagery rescripting and eye movement desensitisation and reprocessing for treatment of adults with childhood trauma-related post-traumatic stress disorder: IREM study design

**DOI:** 10.1186/s12888-017-1330-2

**Published:** 2017-05-04

**Authors:** Katrina L. Boterhoven de Haan, Christopher W. Lee, Eva Fassbinder, Marisol J. Voncken, Mariel Meewisse, Saskia M. Van Es, Simone Menninga, Margriet Kousemaker, Arnoud Arntz

**Affiliations:** 1 Division of Psychiatry, UWA Medical School, Faculty of Health & Medical Sciences, University of Western Australia, 35 Stirling Highway, Crawley, WA 6009 Australia; 2 Lübeck University, School of Psychiatry, Ratzeburger Allee 160, 23562 Lübeck, Germany; 3 Maastricht University, Faculty of Psychology and Neuroscience, Department Clinical Psychological Science, P.O. Box 616, 6200 MD Maastricht, The Netherlands; 4 GGZ Noord-Holland Noord, Stationsplein 138, 1703 WC Heerhigowaard, Netherlands; 5 PsyQ Amsterdam, George Westinghousestraat 2, 1097 BA Amsterdam, Netherlands; 6 PsyQ Beverwijk, Leeghwaterweg 1A, 1951 NA Velsen-Noord, Netherlands; 7 Sinai Centrum, Laan van de Helende Meesters 2, Postbus 2063, 1180 EB Amstelveen, Netherlands; 8 Department of Clinical Psychology, University of Amsterdam, Weesperplein 4, 1018 XA Amsterdam, Netherlands

**Keywords:** Post-traumatic stress disorder, Childhood, Imagery rescripting, Eye movement desensitisation and reprocessing, Treatment

## Abstract

**Background:**

Post-traumatic stress disorder (PTSD) that originates from childhood trauma experiences can develop into a chronic condition that has lasting effects on an individual’s functioning and quality of life. While there are evidence-based guidelines for treating adult onset PTSD, treatments for adults with childhood trauma-related PTSD (Ch-PTSD) are varied and subject to ongoing debate. This study will test the effectiveness of two trauma-focused treatments, imagery rescripting (ImRs) and eye movement desensitisation and reprocessing (EMDR) in participants with Ch-PTSD. Both have been found effective in treatment of adult PTSD or mixed onset PTSD and previous research indicates they are well-tolerated treatments. However, we know less about their effectiveness for treating Ch-PTSD or their underlying working mechanisms.

**Methods:**

IREM is an international multicentre randomised controlled trial involving seven sites across Australia, Germany and the Netherlands. We aim to recruit 142 participants (minimum of *n* = 20 per site), who will be randomly assigned to treatment condition. Assessments will be conducted before treatment until 1-year follow-up. Assessments before and after the waitlist will assess change in time only. The primary outcome measure is change in PTSD symptom severity from pre-treatment to 8-weeks post-treatment. Secondary outcome measures include change in severity of depression, anger, trauma-related cognitions, guilt, shame, dissociation and quality of life. Underlying mechanisms of treatment will be assessed on changes in vividness, valence and encapsulated belief of a worst trauma memory. Additional sub-studies will include qualitative investigation of treatment experiences from the participant and therapists’ perspective, changes in memory and the impact of treatment fidelity on outcome measures.

**Discussion:**

The primary aims of this study are to compare the effectiveness of EMDR and ImRs in treating Ch-PTSD and to investigate the underlying working mechanisms of the two treatments. The large-scale international design will make a significant contribution to our understanding of how these treatments address the needs of individuals with Ch-PTSD and therefore, potentially improve their effectiveness.

**Trial registration:**

Australian New Zealand Clinical Trials Registry ACTRN12614000750684. Registered 16 July 2014.

**Electronic supplementary material:**

The online version of this article (doi:10.1186/s12888-017-1330-2) contains supplementary material, which is available to authorized users.

## Background

Trauma-focused cognitive behaviour therapy (Tf-CBT) and eye movement desensitisation and reprocessing (EMDR) have been identified as the most efficacious post-traumatic stress disorder (PTSD) treatments [1]. Compared to other treatment modalities such as pharmacotherapy, and to non-trauma-focused approaches, EMDR and Tf-CBT interventions have been identified as more effective at reducing PTSD symptom severity [2, 3]. However, the studies from which these treatment recommendations were based, predominantly used samples with adult-onset PTSD, resulting in an underrepresentation of participants with more complex presentations [4, 5]. The research on treatments for one such group, adults with childhood trauma-related PTSD (Ch-PTSD), has suggested that this population is difficult to treat due to the additional symptom complexity that can develop as a consequence of early trauma experiences [6, 7].

Three main approaches for treatment of Ch-PTSD were outlined by Ehring and colleagues [4]. One approach suggests that the aim of treatment should be on improving functioning through skill building, rather than focusing on trauma reprocessing [8, 9]. Secondly, there is the phase-based approach which incorporates skill building with trauma reprocessing techniques such as prolonged exposure [10, 11]. The third approach uses trauma-focused treatments without any modifications to protocol [12, 13]. Treatments are categorised as trauma-focused when they specifically target processing of trauma memories and their meaning [2, 14].

A meta-analysis on psychological treatments for Ch-PTSD, identified individual trauma-focused treatments as being more efficacious than non-trauma-focused approaches [4]. Individual trauma-focused treatments were found to be more effective in reducing PTSD symptom severity and additional symptoms associated with Ch-PTSD such as depression, anxiety and dissociation. Moreover, the findings also supported the view that increased levels of symptom complexity are probably not a contraindication for trauma-focused treatment. Nevertheless, there is reluctance by many therapists to engage in trauma-focused treatments as a first-line approach for individuals with Ch-PTSD [15, 16].

There is much debate regarding the appropriateness of trauma-focused interventions and some have argued that treatments which primarily focus on trauma reprocessing, are inappropriate for the Ch-PTSD population [17, 18]. The argument is mostly based on the perception that individuals with Ch-PTSD only have a limited capacity to cope with the distress of focusing on their trauma experiences during treatment, while remaining physically, psychologically and emotionally intact [9]. Indeed, some studies have reported dropouts rates of up to 41% e.g. McDonagh et al. [19], which many attribute to the more complex symptom presentations often found in individuals with Ch-PTSD [8, 17]. However, these findings have predominantly been in studies that incorporated prolonged exposure as a component of treatment [19, 20]. This suggests the need to explore alternative trauma-focused approaches for treatment of Ch-PTSD.

Other trauma-focused treatments such as EMDR and Imagery Rescripting (ImRs) have been proposed for Ch-PTSD [21, 22]. EMDR and ImRs share similarities with prolonged exposure techniques by activating imagery, emotions and cognitions related to the trauma memory and providing corrective information. The difference with these treatments is that they do not require intensive and prolonged reliving of traumatic experiences to enact symptomatic reductions [23, 24].

EMDR asks individuals to recall their trauma experience in their mind while at the same time tracking the back and forth movement of the therapist’s finger [25, 26]. This dual attention focus facilitates reconsolidation of the original trauma memory so that it is less vivid and less distressing [23]. The precise mechanism by which EMDR appears to facilitate trauma processing is unknown [27]. There have been several theories proposed, however a more recent explanation for the underlying mechanisms of EMDR, which has received the most empirical support, is the working memory theory [28]. This theory postulates that eye movements tax the capacity of the working memory, thereby making the trauma memory less vivid and consequently, difficult for individuals to maintain the traumatic experience with the same degree of emotional distress [29, 30].

Given that EMDR is an evidenced based treatment for PTSD, surprisingly there are only a limited number of studies examining its effectiveness for treating Ch-PTSD [12, 31]. In one study of adults who had been sexually abused as children, EMDR significantly reduced PTSD symptom severity at post-treatment and at 18-month follow-up [22]. Another study of traumatised young women found both the EMDR and active listening conditions resulted in significantly improved scores on measures of PTSD, depression, anxiety and self-concept. More interestingly, the EMDR group improved to within one standard deviation of the mean of the general population norms on all measures and the pre-post treatment effect sizes were almost double that of the active listening condition [32]. Thus, in the context of Ch-PTSD, EMDR could make a significant impact on individuals’ capacity to tolerate exposure to traumatic material and potentially improve treatment outcomes [22, 33].

ImRs involves the individual imagining a different ending to a trauma experience. Individuals are encouraged to recall a memory in the first person, present tense as their child self. The memory is then rescripted, by imagining a different course of events, which helps to satisfy the needs of the person [24, 34]. ImRs aims to facilitate a change in the meaning or reinterpretation of the trauma memory, leading to fundamental shifts in core belief systems and behaviours; and also provides an opportunity for individuals to identify and express responses that were inhibited at the time of the trauma experience [24, 35]. ImRs might be especially suitable for treating Ch-PTSD. For example, it is shown to be particularly efficacious for interpersonal traumas where trust was violated, and it has been shown as more effective than imaginal exposure in treating not just anxiety but other emotions such as guilt or shame, all of which are common in childhood trauma-related PTSD [21, 36].

ImRs research is still in its infancy and there is limited understanding for the underlying mechanisms of this treatment. The predominant explanation is that ImRs works by changing the meaning of trauma event [21, 35]. Arntz [21, 36] postulated that a process called unconditioned stimulus-revaluation might underlie ImRs. That is, after the trauma experience is consolidated in memory it is possible to reactivate the experience during ImRs. During the rescripting procedure the memory is re-evaluated so that its dysfunctional meaning changes to a less dysfunctional association, after which the representation is re-consolidated. This leads to less negative-emotional responses when the memory is activated in future. Dibbets and colleagues [37] tested this hypothesis in a classical conditioning study and found evidence that ImRs reduced the return of fear, which usually occurs when an individual is retriggered with the context associated with the acquisition phase. The observed reduction in return of fear is an indication that the unconditioned stimulus-representation fundamentally changed as to its fear-evoking meaning thru the ImRs procedure. In contrast, a recent study by Slofstra, Nauta, Holmes and Bockting [38] reported that a specific focus on manipulating the semantic meaning-relevant content of trauma memories, that is key cognitions or beliefs, was not necessary for ImRs to be effective. Instead, when rescripting focused on only perceptual aspects of trauma experiences, ImRs facilitated reconsolidation of memories and associated emotions.

Notwithstanding, the growing evidence suggests ImRs is an efficacious treatment [21, 39]. Recent research has also supported its effectiveness in treating complicated PTSD. In one such study ImRs was used to treat refugees and the treatment was found to lead to significant reductions in PTSD symptom severity and depression scores [40]. More specifically, a recent study for adults with childhood abuse-related PTSD showed that ImRs decreased PTSD related symptoms, all of which were maintained or improved at follow-up assessment. In addition, the dropout was 20%, which could be considered low in comparison to other trauma-focused interventions [41].

ImRs in the context of adverse childhood memories has been successfully applied to the treatment of disorders such as social anxiety, simple phobias, bulimia nervosa and depression, showing good treatment effects [39]. Taken together, the evidence supports ImRs as an efficacious treatment for PTSD and for addressing aversive childhood memories.

To date there has only been one study that has directly compared ImRs and EMDR. Alliger-Horn, Zimmermann and Mitte [42] compared EMDR and imagery rescripting and reprocessing therapy, a variant of ImRs, in the treatment of soldiers with PTSD. This study reported significant improvements in post-traumatic and comorbid symptoms for both treatments with no significant differences between the two. However, the sample size was small (20 in each condition) and the participants treated had PTSD from adult trauma experiences.

There are theoretical reasons and some preliminary findings to suggest EMDR and ImRs are approaches worthy of further investigation [21, 33]. Furthermore, different working mechanisms having been proposed for the two treatments, the reduction of vividness of trauma memories as a primary mechanism in EMDR versus the change in (emotional) meaning of the trauma memory in ImRs. A direct comparison of the two treatments with repeated assessments of vividness, valence, and encapsulated beliefs of a trauma memory will enable a better understanding of underlying mechanisms of action and potential differences of change between the two approaches.

Nevertheless, research interest is growing. However, clinical trials investigating treatments for Ch-PTSD, more specifically trauma-focused approaches are scarce [4]. Some have argued that the reluctance of therapists to directly target trauma experiences as a first-line intervention approach, has led to individuals being denied or delayed from receiving appropriate treatment [14, 20]. These factors together highlight the need for investigating treatments, which effectively treat symptoms of Ch-PTSD and are acceptable to individuals and therapists.

### This study

This article describes the study design of IREM, an international, multi-centre randomised clinical trial (RCT) whose primary objectives are to compare the effectiveness of EMDR and ImRs in the treatment of Ch-PTSD and to test whether different mechanisms of change are involved.

A number of additional sub-studies will be performed which include the investigation of participant and therapists’ perspectives of treatment, assessing the effects of treatment integrity, prediction of drop out and changes in how trauma memories are stored following treatment.

## Method

### Design

An international multicentre RCT will be conducted in Australia, Germany and the Netherlands, see Additional file 1: Appendix 1. Seven sites will participate with one site in Perth, Western Australia; one in Lübeck, Germany; and five in the Netherlands. Ethical approvals were obtained by ethics committees in each country. This trial is registered with the Australian and New Zealand Clinical Trials registry, ACTRN12614000750684 and complies with the World Health Organization Trial Registration Data Set, see Additional file 1: Appendix 2. This RCT adheres to the SPIRIT guidelines and methodology.

### Recruitment

**Fig. 1:**
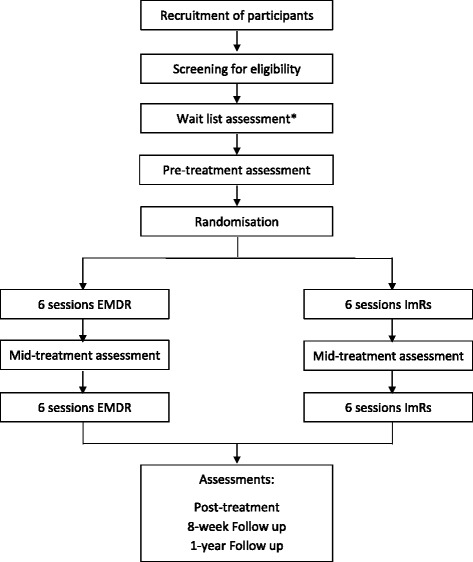
IREM design flow chart. * Waitlist assessment included for sites that have a waitlist of less than 3 weeks before the start of treatment

Potential participants will be identified and screened at each site when childhood trauma-related PTSD is suspected. Participants will attend an information session where they will be provided verbal and written information of the research project. If they agree to participate they will provide signed informed consent and be formally screened to assess in- and exclusion criteria, see Additional file 1: Appendix 3. Figure 1 provides an overview of the study design.

### Participants

Male and females aged between 18 and 70 years of age will be considered eligible if 1) they experienced trauma before 16 years of age and agree that this will be the focus of treatment, 2) PTSD is the primary diagnosis, 3) PTSD symptoms are present for longer than 3 months and mainly associated to the trauma that occurred before the age of 16, 4) they are available to attend treatment sessions twice a week within a 6 to 8 week period and 5) they are able to understand, read, write and speak the language of the site or English where sites permit. If participants are on any psychological medication it is required the dose has been stable for 3 months. The participants have to agree not to engage in any other psychological therapy or have changes in medication from baseline until the 8-week post-treatment assessment.

Participants will be excluded if they have comorbid psychotic disorder, bipolar disorder type 1, alcohol or drug dependence, IQ below 80, acute suicide risk, acute PTSD from trauma within the past 6 months, PTSD focused treatment within the past 3 months or scheduled to begin another form of PTSD treatment. Current benzodiazepine use is also an exclusion criterion. However, a participant is eligible to participate if they agree to taper off their benzodiazepine medication for their involvement in the study including an additional 2-week period before they may begin the assessment process.

### Sample size

At least 142 participants across all sites will be recruited for the RCT. Each site is required to recruit a minimum of 20 participants.

To achieve a between group medium effect size using Cohen’s *d*, with a significance level of .05 (two-tailed), it would require a sample size of *N* = 128. To account for estimated dropouts of 10%, the sample size is increased to *N* = 142. This number is the total across the sites involved in this research. With the use of mixed regression, taking into account all available data, the actual power will be higher and the standard error will be reduced with the use of covariates.

### Randomisation

An independent central research assistant will randomise participants to treatment condition after checking inclusion and exclusion criteria. Randomisation will be based on block randomisation (n = two, four and six per block, with block size randomised) per site, to guarantee a balance between conditions per site and over time. Randomisation will be stratified for gender to control distribution per treatment at each site.

### Treatments

Treatment will consist of 12, 90-min sessions twice a week. Treatment is scheduled for completion within a 6-week period, however up to 8 weeks is permitted. Participants are allowed to complete treatment earlier than the allocated 12 sessions, however assessment will be conducted at the planned time points. Earlier termination of treatment requires approval from individual site coordinators.

#### Treatment procedure

Session one of the treatment will involve introduction to treatment rationale and planning of treatment sessions. The therapist and participant will develop a record of trauma memories to be targeted during treatment and agree on which memory will be addressed first. The record of trauma memories will be given to the participant to review before the next session.

In the ImRs condition, a pilot rescripting will be conducted in session one so that participants are familiar with the technique. For the EMDR protocol, due to time constraints, there will be no pilot in the first session. Within this condition, the therapist will focus on preparing for processing in session two.

At the beginning of session two, the therapist and participant discuss any changes to the record of trauma memories that were identified. From treatment session two onwards, each session requires trauma reprocessing in the allocated treatment condition.

Treatment sessions are video or audio recorded depending on the site and participant consent. The duration of the session and the number of trauma memories that were addressed during each treatment session are recorded.

The EMDR and ImRs treatments have been operationalized into manuals.

#### Eye movement desensitisation and reprocessing (EMDR)

EMDR treatment is based on the eight-phase protocol outlined by F Shapiro [25]. Session one in the EMDR treatment condition will involve procedural preparation and affect tolerance training including a safe place exercise, which is practiced between sessions. The therapist and participant will set up for processing in session two by completing up to phase three of the EMDR protocol (target assessment), and within that only as far as identifying the negative and positive cognition.

Desensitisation will begin in session two where the participant will be asked to focus on the memory, their negative belief, feelings and somatic sensations while they follow the back and forth movement of the therapist’s fingers. Sets continue until the reported distress is decreased to O or 1. In the installation phase, the participant is asked to focus on the preferred belief (positive cognition) and the trauma memory while simultaneously engaged in the eye-tracking task. As per the F Shapiro [25] protocol, once the positive cognition is reported as 6 or 7, a body scan is conducted to ensure no unresolved traumatic material. Each session concludes with a debriefing of participants. At the start of each session, the therapist would check processing of the memory that was targeted during the previous session. If the distress reported is 2 or more, processing would continue on the memory. Alternatively the participant and therapist will select another target memory for processing. In session 12, the therapist would evaluate any current triggers or future oriented events that would need to be desensitised; this may be completed earlier if all trauma memories have been reprocessed in earlier sessions.

Some modifications have been made to the original EMDR protocol to take into account recent literature, e.g. no eye movements in the safe place protocol [43]. In addition, a restriction has been placed on types of unblocking strategies particularly those using imagery during the interweave to prevent contamination of the treatment condition.

#### Imagery rescripting (ImRs)

The ImRs treatment is based on the protocol described by A Arntz and A Weertman [34]. During the rescripting participants are asked to describe the trauma memory in first person, present tense, from the point of view of the child, what is happening in the image, what they are thinking, feeling and most importantly, what they need. They are then guided to imagine a different course of events that is more acceptable for meeting their needs. The first half of ImRs treatment, up to session six, the therapist steps into the image and intervenes, protects and meets the needs of the child. In later phases, from session seven onwards, the participant’s adult self steps into the image and then rescripts from the point of view of the adult self. Next the participant re-experiences the rescripting from the point of view of the child, with the adult intervening. The child is then invited to ask their adult self for additional actions if there is anything else they would like until all needs have been met.

After each rescripting, the therapist and participant debrief to ensure that all the needs were met. Trauma memories will be rescripted until the participant is satisfied. More than one trauma memory can be rescripted during a treatment session.

#### Further treatment

Evaluation of participants need for further treatment is conducted after the 8-week post-treatment assessment. The kind, intensity and frequency will be determined based on needs of the participant and the capacity of the site. Details of any further treatment will be documented and reported.

### Therapists, training and supervision

The therapists in this study will be licensed psychologists (including clinical and health), psychotherapists, psychiatrists and a psychiatric nurse with advanced mental health qualifications. Dependent on the site, therapists will be trained in one or both treatment conditions. EMDR therapists must have completed EMDR training level 1 with an additional 2-day training for Ch-PTSD for the present study. Chris Lee an approved EMDR International Association trainer, provided training in the EMDR protocol across each site. ImRs therapists must have completed basic training in CBT and an additional 2-day training for Ch-PTSD for the present study. Arnoud Arntz provided ImRs training for each site. Before commencing treatment with study participants, therapists’ are required to demonstrate they are competent to apply the treatment with a minimum of two pilot cases that they video record and show to the peer supervision group and site coordinator. In addition, therapists are required to attend 5 h of peer supervision before starting with study participants and commit to having regular peer supervision throughout the course of the study. Where there is concern regarding therapists’ competence or issues relating to treatment protocol, expert supervision will be available from the aforementioned EMDR and ImRs trainers.

### Measures

#### Primary outcome measures

##### The Clinician Administered PTSD Scale for DSM-5 (CAPS-5; [44])

The CAPS-5 is a structured interview consisting of 30-items to assess PTSD symptoms over the previous month. Research assistants are required to have training and supervision in the use of the CAPS-5 before conducting assessments with study participants.

As the CAPS-5 measures symptoms over the past month, post-treatment assessment would not be reflective of clinical improvement from the 12 intervention sessions as the assessment period overlaps with treatment. Therefore, the primary outcome measure is change in PTSD symptom severity measured by the CAPS-5 comparing pre-treatment to the 8-week post-treatment assessment and pre-treatment with follow-up.

#### Secondary outcome measures

##### The Impact of Events Scale – Revised (IES-R; [45])

The IES-R is a 22-item self-report questionnaire measuring symptomatic response over the last 7 days, to a specified trauma event. Four items were added to the IES-R to assess trauma-related guilt, anger, disgust, and shame, see Additional file 1: Appendix 4 [24]. Therapists are recommended to use these ratings to guide treatment sessions.

##### The Beck Depression Inventory II (BDI-II; [46])

The BDI-II is a 21-item self-report instrument assessing depressive symptoms during the last 2 weeks.

##### The Post-Traumatic Cognitions Inventory (PTCI; [47])

The PTCI is a 33-item self-report instrument used to assess cognitions considered to underlie posttraumatic psychopathology.

##### The Trauma-Related Guilt Inventory (TRGI; [48])

The TRGI is a 32-item self-report questionnaire, which measures cognitive and affective aspects of trauma-related guilt.

##### The Trauma-Related Shame Inventory (TRSI; [49])

The TRSI is a 24-item self-report instrument to assess individual’s negative self-evaluations in the context of their traumatic experiences.

##### The Anger Expression and Control Scale (ZECV; [50])

The ZEVC is a 40-item scale to assess internalised and externalised anger.

##### The hostility subscale of the Symptom Checklist-90-R (SCL-90-R; [51])

The hostility subscale of the SCL-90-R is a 6-item scale to assess anger related thoughts, feelings and behaviours.

##### The Dissociative Experiences Scale-Taxon (DES-T; [52])

The DES-T is an 8-item scale designed to measure symptoms of pathological dissociation.

##### The Happiness Questionnaire (HQ [53])

A single item question will be used to assess overall level of happiness with life.

##### The Remoralization Questionnaire (RQ; [54])

The RQ is a 12-item questionnaire used to assess restoration of morale that is considered to be important in the process of therapeutic change.

##### The Schema Mode Inventory (SMI; [55])

The SMI is a 118-item scale used to explore schema modes. The SMI will be assessed in all sites excluding Perth as recruitment had already commenced at this site prior to the inclusion of this measure.

##### Imagery Interview (II; [28, 56–58])

An imagery interview will be used to assess memory vividness, memory distress, and encapsulated belief by having the participants rate these aspects on a 0–100% scale immediately after imagining their memory of the index trauma.

##### The World Health Organization Disability Assessment Schedule 2.0 (WHODAS; [59])

The WHODAS is 15-item questionnaire designed to measure the levels of functioning of an individual across major life domains including cognition, self-care, daily activities, mobility, social interaction and community participation.

##### Medication Use (Medication)

Medication use will be assessed at each assessment and treatment session and will collect details on any changes in medication related to psychological disturbances.

### Study procedures

#### Screening procedure

The screening session will assess potential participants eligibility based on the aforementioned in- and exclusion criteria and their motivation for trauma-focused treatment. Psychiatric disorders will be assessed with the Structured Clinical Interviews for DSM-IV-TR (SCID; [60]) or the Mini International Neuropsychiatric Interview (MINI; [61]), depending on site preference.

If study criteria have been satisfied, trauma history will be assessed using the Life Events Checklist for DSM-5 (LEC-5), a 17-item self-report questionnaire developed to screen for lifetime trauma experiences [62]. Additional items have been added to the LEC-5 to assess emotional abuse/neglect and physical neglect, see Additional file 1: Appendix 5. The LEC-5 will be administered during the screening session to identify the nature and extent of trauma experiences.

To determine the focus of assessments, during the screening session participants will be asked to identify 1) an *index trauma*, being a single or group of closely related events for example ‘the sexual abuse by my uncle’, that was experienced before 16 years of age which will be the traumatic event (PTSD criterion A) for the CAPS-5 clinical interview; 2) a *worst memory* related to the index trauma and associated encapsulated belief that will be used for the imagery interview.

#### Assessment procedure

Assessments will be conducted by a research assistant who is blind to treatment condition. Assessments are all audio recorded and are a combination of clinical interview and self-report instruments.

There will be either five or six assessment sessions during the study, see Table 1. Assessments will be conducted at waitlist (if there is less than a 3-week gap between waitlist and commencement of treatment), pre-treatment, mid-treatment (after 6 sessions), post-treatment (8 weeks after the first treatment session), 8-week follow-up (8 weeks after the post-treatment assessment) and 1-year follow-up (1 year after pre-treatment assessment). Participants who have successfully completed treatment earlier than the possible 12 sessions will be assessed at the planned assessment moment.

**Table 1:** Overview of measures and assessment times

	Screening	Baseline	Pre-treatment	Mid-treatment	Post-treatment	8 week follow up	1-year follow up
SCID or MINI	•						
LEC-5	•						
Demographics		•					
Imagery interview		•	•	•	•	•	•
CAPS-5		•	•		•	•	•
WHODAS		•	•		•	•	•
Medication^a^		•	•	•	•	•	•
Happiness		•	•		•	•	•
IES-R^a^		•	•	•	•	•	•
BDI-II		•	•		•	•	•
DES-T		•	•	•	•	•	•
PTCI		•	•	•	•	•	•
TRGI		•	•	•	•	•	•
TRSI		•	•	•	•	•	•
ZECV		•	•	•	•	•	•
SCL-90-R		•	•	•	•	•	•
RQ		•	•		•	•	•
SMI^b^		•	•		•	•	•

*SCID* Structured Clinical Diagnostic Interview, *MINI* Mini Neuropsychiatric Interview, *LEC-5* Life Events Checklist, *CAPS-5* Clinician Administered PTSD Scale, *WHODAS* World Health Organisation Disability Assessment Schedule, *IES-R* Impact of Events Scale – Revised, *BDI* Beck Depression Inventory, *DES-T* Dissociative Experiences Scale-Taxon, *TRGI* Trauma-Related Guilt Inventory, *TRSI* Trauma-Related Shame Inventory, *ZECV* Anger Expression and Control Scale, *SCL-90-R* Hostility subscale of the Symptom Checklist Revised, *RQ* Remoralization Questionnaire, *SMI* Schema Mode Inventory. ^a^IES-R to be completed by participant and medication use to be recorded, at the start of each treatment session. ^b^SMI to be completed for each site excluding Perth, Australia.

Self-reported PTSD symptoms using the IES-R will be assessed twice at each assessment and treatment session, once for the ‘Index trauma’ and again for ‘all others traumas’ as identified on the LEC-5. The IES-R administered at session seven will be used as the mid-treatment assessment.

### Analysis

Taking into account all the data collected, a mixed regression model will be used to analyse the data. This will identify any fixed or random effects resulting from the treatment and changes over time. A mixed logistic regression analysis will be used for diagnostic outcomes. A mixed gamma regression will be used for skewed distributions. Poisson or negative binomial regression will be used for analysing medication use and other count data. Analysis will include an intent-to-treat sample.

The mechanism test will be done by advanced mediation tests, using multiple assessments of dependent variables (CAPS-5, IES-R) and variables representing indices that should predict change in symptoms (imagery interview-based ratings of vividness, valence, and encapsulated beliefs of the primary trauma memory).

## Additional sub-studies

Secondary investigations will be performed to explore and further our understanding of the effectiveness of EMDR and ImRs in the treatment of Ch-PTSD.

### Treatment

#### Treatment integrity

Independent raters blind to treatment condition will rate a random selection of session recordings to assess treatment integrity. Ratings will be used to document treatment integrity and to investigate the relationship with treatment outcome.

#### Prediction of effects and drop out

Baseline characteristics of participants such as symptom severity and nature of trauma will be used to study whether they predict treatment effects and dropout, or if such variables predict a different response to EMDR versus ImRs.

### Mechanisms and essential ingredients of treatments

#### Participants’ views on central mechanisms of EMDR and ImRs – qualitative

The objective of this study is to get a better overview of what participants consider the most effective elements of EMDR and ImRs treatments. Qualitative interviews will be conducted to explore if participants experienced changes related to the techniques and in what areas the changes were experienced.

#### Essential ingredients of ImRs – Observational study

The purpose of this study is to clarify the working mechanisms of the ImRs protocol, in order to enlarge the chance of a successful treatment of PTSD-symptoms for participants with early childhood trauma. This study will use the video recordings of ImRs sessions to explore on a microscopic, observational level what specific ingredients of ImRs are associated with change, with a specific focus on two possible processes: expression of inhibited action tendencies and need fulfilment.

### Participant and therapist perspectives of treatment

The exploration of the treatment experience and factors related to treatment will further our knowledge of issues and barriers associated with the implementation of Ch-PTSD treatments. These investigations will be a mixed methods approach. Therapists involved in this study will complete an online survey to explore attitudes, beliefs and experience of treating Ch-PTSD. Following this, further exploration into therapists’ perspectives will be conducted with in-depth interviews. Questions for these qualitative interviews will focus on experience of delivering treatment, training, supervision, challenges and opinions.

Qualitative interviews will be performed to explore the treatment experience from the participants’ perspective. This study will aim to investigate the participant’s experience of treatment, the process of change and factors related to treatment engagement such participant motivation and difficulty during treatment.

### Changes in memory

#### Change in specificity of memories

This study will aim to test whether memories of single trauma are more specific and consistent than those of repeated traumas. Participants are asked to write an account of the index trauma and where there are multiple traumas that constitute the index trauma, to describe the one they have the clearest memory of, at pre-treatment and again at the 8-week post-treatment assessments.

#### Change in memory consolidation

The aim of this study is to explore the nature of change in memories of traumatic events, with a view to identifying factors that are associated with successful treatment outcomes. This study is a qualitative design that will compare transcripts of participants’ pre- and post-treatment index trauma memories using a coding system informed by the literature base and clinical expertise.

## Discussion

This article has described the study design of IREM, an international multicentre RCT comparing the effectiveness of EMDR and ImRs for treatment of Ch-PTSD. This RCT and the additional sub-studies will broaden our understanding of the effectiveness of these approaches in Ch-PTSD treatment and will help in furthering our understanding of their underlying mechanisms of change.

The study design has several strengths. Given the RCT is an international multi-site design, conclusions about any treatment effects will be generalisable to the method, rather than idiosyncratic ways a treatment might be delivered in a particular country or a particular site. In addition, this RCT has scientific validity as all the measures are standardised and assessments will be conducted by researchers who are blind to treatment condition. Another strength is that there are few exclusion criterion, thus enhancing the generalisability of the findings to real world settings. The overall size of the sample should provide sufficient power to enable meaningful conclusions about the data.

A limitation of the study is that there is no randomization between waitlist and active treatment. Instead, a naturalistic waitlist will be used to test whether treatment differs from natural course, assuming that whether or not patients have to wait until they can start treatment is nonbiased, as it is driven by the site’s treatment capacity at the moment. To prevent bias, no patient will be given priority to start treatment. Another limitation is that there is no budget for intensive treatment supervision by experts. On the other hand, the study will document what the effectiveness is of the treatments based on a simple dissemination program. Relatedly, the therapist requirements differ between conditions: for EMDR therapists are required to complete a minimum level one training; whereas for ImRs only a basic CBT training is required. The authors feel that this adequately represents reality with EMDR perhaps being more complex to train than ImRs.

The evidence from this RCT will contribute to clinical decisions on whether to use trauma-focused approaches for the sequela associated with Ch-PTSD. While the primary aims of this study is to compare EMDR and ImRs treatments on PTSD symptom severity and to clarify whether or not different mechanisms of change are involved, secondary outcome measures will assess some disturbances associated with Ch-PTSD such as guilt, shame, anger and dissociation. EMDR and ImRs might more directly target change through cognitive and experiential mechanisms, thus having a greater impact on a wider range of symptoms than just PTSD [21, 63].

This study will contribute to the growing evidence for the efficacy of trauma-focused treatment for Ch-PTSD. The documented lack of implementation of trauma-focused approaches has highlighted the need to explore treatments, which will be acceptable to both participants and therapists [6, 20]. A large part of the issue for treatment of Ch-PTSD is the perception that individuals would not be able to tolerate treatment. However, it is unclear if the reluctance to engage in trauma-focused treatment is a shared view as it is suggested that avoidance of the therapist is a much greater barrier [20]. This RCT will be able to contribute to our knowledge of participants’ capacity to engage in treatment. The exploration into therapist and participants’ perspective will help to identify barriers and issues with the implementation of treatment.

Taken together, findings from this RCT will make a significant contribution towards developing best practice for treating PTSD caused by childhood experiences. Both EMDR and ImRs hold promise as being efficacious treatments for Ch-PTSD and the sequela of symptoms associated with this disorder.

## Additional file

Additional file 1: Appendix 1. Mutual Agreement IREM Study. The mutual agreement endorsed by each of the participating sites outlining the roles and responsibilities of each university representative and site coordinator. The mutual agreement also includes the study timeline and planned publications. **Appendix 2.** World Health Organization Trial Registration Data Set. WHO Trial Registration Data Set completed to support SPIRIT Guidelines Checklist. **Appendix 3.** Consent form example. An example of the consent form that will be given to participants to read and sign to ensure informed consent. **Appendix 4.** Additional items added to the Impact of Events Scale Revised (IES-R). Four items that were added to the IES-R to assess trauma-related guilt, anger, disgust, and shame. **Appendix 5.** Additional items added to the Life Events Checklist (LEC-5). Additional items have been added to the LEC-5 to assess emotional abuse/neglect and physical neglect. (DOCX 136 kb)

## Abbreviations

BDI-II: Beck Depression Inventory
CAPS-5: Clinician Administered PTSD Scale for DSM-5
Ch-PTSD: Adults with childhood trauma-related PTSD
DES-T: Dissociative Experiences Scale-Taxon
DSM-5: Diagnostic and statistical manual of mental disorders
EMDR: Eye movement desensitisation and reprocessing
HQ: Happiness question
IES-R: Impact of Events Scale – Revised
II: Imagery interview
ImRs: Imagery rescripting
LEC-5: Life Events Checklist
MINI: Mini Neuropsychiatric Interview
PTCI: Post-Traumatic Cognitions Inventory
PTSD: Post-traumatic stress disorder
RCT: Randomised controlled trial
RQ: Remoralization Questionnaire
SCID: Structured Clinical Diagnostic Interview
SCL-90-R: Hostility subscale of the Symptom Checklist Revised
SMI: Schema Mode Inventory
Tf-CBT: Trauma-focused cognitive behaviour therapy
TRGI: Trauma-Related Guilt Inventory
TRSI: Trauma-Related Shame Inventory
WHODAS: World Health Organisation Disability Assessment Schedule
ZECV: Anger Expression and Control Scale
