# Supplementary material for: Imagery rescripting and eye movement desensitisation and reprocessing for treatment of adults with childhood trauma-related post-traumatic stress disorder: IREM study design
Source: BMC Psychiatry. 2017 May 4;17:165. doi: 10.1186/s12888-017-1330-2 (PMC5418842; doi:10.1186/s12888-017-1330-2)
Supplement: Additional file 1: Appendix 1. — Mutual Agreement IREM Study. The mutual agreement endorsed by each of the participating sites outlining the roles and responsibilities of each university representative and site coordinator. The mutual agreement also includes the study timeline and planned publications. Appendix 2. World Health Organization Trial Registration Data Set. WHO Trial Registration Data Set completed to support SPIRIT Guidelines Checklist. Appendix 3. Consent form example. An example of the consent form that will be given to participants to read and sign to ensure informed consent. Appendix 4. Additional items added to the Impact of Events Scale Revised (IES-R). Four items that were added to the IES-R to assess trauma-related guilt, anger, disgust, and shame. Appendix 5. Additional items added to the Life Events Checklist (LEC-5). Additional items have been added to the LEC-5 to assess emotional abuse/neglect and physical neglect. (DOCX 136 kb) [file 12888_2017_1330_MOESM1_ESM.docx]

IREM Study Protocol – Appendix

Appendix 1. Mutual Agreement IREM Study

Mutual Agreement IREM study

Between

**NAME** institute of Mental Health at **CITY** in **COUNTRY**

and

the study organization formed by Maastricht University (Dr. Marisol Voncken), University of Amsterdam (Dr. Arnoud Arntz), Murdoch University (Dr. Chris Lee) and University of Lübeck (Dr. Eva Fassbinder), represented by the principal investigator (Dr. Arnoud Arntz).

The **NAME** institute of Mental Health at **CITY** in **COUNTRY** agrees to participate in the international multicenter study on the effectiveness, working mechanisms and stakeholders’ perspectives of Imagery Rescripting vs. EMDR for PTSD related to trauma that happened before the age of 16, hereafter called the IREM study. More specifically, the institute will recruit the necessary PTSD patients (N=20), that fulfill the in/exclusion criteria of the study; the institute will not keep potential or actual participants of the study from the study; will accept randomization outcome; and will deliver Imagery Rescripting (ImRs) and EMDR as described in the treatment protocols, given by qualified therapists, as stipulated in the research protocol. In case patients are randomized to EMDR, therapists will follow the directions given by the study’s EMDR supervisor Dr. Chris Lee, or any other study’s EMDR supervisor appointed by the study board, unless legal or clinical issues prevail. In case patients are randomized to ImRs, therapists will follow the directions given by the study’s ImRs supervisor Dr. Arnoud Arntz, or any other study’s ImRs supervisor appointed by the study board, unless legal or clinical issues prevail. The supervisors will also have a final say on whether therapists qualify as EMDR-therapists, respectively ImRs-therapists, or not. For all research issues, the institute will follow the directions of the primary investigator (PI), Dr. Arnoud Arntz, unless legal or clinical issues prevail.

The institute and its employees will also follow all the study and study-treatment requirements not explicitly listed here but described in the study protocol and the study’s EMDR respectively ImRs treatment protocols. In case of issues that cannot be resolved by mutual agreement in the Study Board, the primary investigator decides, unless legal or clinical issues prevail.

The institute will cover the costs of the treatment in both conditions including crisis intervention if necessary and the cost of psychiatrists seeing patients for medication related issues or coordinating inpatient hospitalization, etc. The institute will further cover the costs of local data collection and storage, of therapists, research assistants and local coordinator.

The study organization will cover the following costs and work: ethical screening of the (sub)study; the training of therapists (excluding the therapists’ salary, travel and hotel costs); central supervision (via internet); central data management and internet applications for automatic administration of questionnaires and interview data; central costs of analyzing and publishing the data. The central organization will provide the therapists with EMDR and ImRs treatment protocols and the institute’s research assistants with the necessary interviews and questionnaires.

The institute is represented in the study’s study board by **NAME** who will be the institute’s local coordinator of the study.

This local coordinator will qualify for (co-) authorship of the publications of the key studies. The institute’s local coordinator or other employees of the institute can qualify for (co-) authorship of publications of ancillary studies, but only if they fulfill American Psychological Association (APA) criteria for authorship. Agreements on this will be made in Study Board meetings and recorded in the overview of planned publications (see Appendix for current overview). The institute will not put any pressure on the local coordinator nor on the Study Board or the PI to have any person of the institute not fulfilling APA criteria for authorship added as (co-) author to publications of the IREM project.

If there are developments which may endanger the study, the institute will warn the primary investigator as soon as possible and start to solve problem thru mutual agreement.

In case of gross negligence to meet what is listed above, the institute loses its rights to deliver a co-author and is obliged to pay a compensation for the costs of therapists’ training on the basis of regular commercial tariffs.

The institute is aware of and agrees with the project management described below.

1. A Study Board acts as the steering committee. It consists of a PI who is the chair (Arnoud Arntz), the other universities’ representatives (Marisol Voncken, Chris Lee, Eva Fassbinder), and one coordinator of each participating mental health institute. Publication agreements and decisions about how the data will be shared are being made in the study board meetings. An addendum lists the current agreement on planned publications related to the main and ancillary studies. All agree that the Study Board owns the data collected with the IREM study. Issues that arise after this agreement is effective will be decided by a majority vote of the study board. If members cannot agree with the Study Board’s majority vote or issues arise that cannot be settled at the Study Board, all parties have agreed that issues that involve human ethics will be referred to the ethical committee that handled the IREM study in their country. Other issues will be referred to the PI’s University.

2. At each institute the local coordinator will be responsible for ensuring that the research plan is followed. The institute gives its local coordinator full mandate to be able to fulfill the this role. The PI and the Study Board can rely on that the local coordinator has the mandate to run the study at the institute.

3. Each institute is responsible for delivering the agreed upon number of subjects in the time that is agreed upon in the Study Board.

4. Research assistants will be assigned by the institute. The institute will guarantee that research assistants get proper training, in collaboration with the study organization, that will organize training of research assistants (including CAPS training). Research assistants will follow the assessment protocol of the study. The research assistants follow the directions of the central research assistant, of the local coordinator, and of the PI. The institute guarantees access to central internet-based data system (Emium) and video-conferencing. The institute organizes video-recordings of treatment sessions and assessments as specified by the study organization, data-storage of these video-recordings, and uploading of requested recording to the central data-storage. The institute organizes peer-supervision for study-therapists as specified in the research protocol, assigns a chairperson of the peer-supervision group, and let the chairperson contact the central supervisor(s) in case of doubt or problems.

The institute’s legal representative:

Name:

Position:

Place:

Date:

Signature:

The institute’s local coordinator, representative in the study board:

Name:

Position:

Place:

Date:

Signature:

On behalf of the study organization:

Arnoud Arntz, PI

University of Amsterdam & Maastricht University,

The Netherlands

Amsterdam,

Date:

Signature:

**Dissemination and Implementation.**

The results of the study will be disseminated in the scientific community by publications in scientific journals and presentations at scientific conferences. Clinicians will be informed by presentations at conferences attended by clinicians (e.g., the national and international conferences), chapters and books describing the protocol (or protocols if treatments don’t differ substantially). Moreover, trainings in the optimal method will be developed and offered to clinicians, as well as supervision in the superior technique. Among participating therapists are teachers (e.g., courses in treatment of (complex) PTSD) and supervisors, which will facilitate dissemination. Implementation will be stimulated by offering in-company training and supervision, and by informing national clinical guideline committees.

**Time schedule**

September – October 2014: first training of therapists and research assistants

March-May 2016 second training of therapists and research assistants

October 2014: start of recruitment of patients, assessment of in/exclusion criteria, first assessments, first randomizations

November - December 2014: start of treatments, peer and specialist supervision, data are centrally stored, checked and prepared for analysis

September 2018: last treatments finish

September 2017- September 2019: Last Follow-Up assessments; analysing of outcome data, reports of results (articles, conferences). Start of dissemination and implementation activities.

Appendix. Current overview of planned publications.

Note: APA-guidelines are decisive.

Key publications / primary studies

1. Design paper
   - Authors: study board members / first: Katrina Boterhoven de Haan
2. Outcome (clinical effectiveness)
   - Authors: study board members / first: to be decided
3. Mechanisms
   - Authors: study board members / first: to be decided

Secondary publications / ancillary studies

1. Participants’ views: overall opinion on and satisfaction with EMDR and ImRs, a qualitative study
   - First author: Katrina Boterhoven de Haan
   - Co-authors: Chris Lee (senior), Helen Correia, Simone Menninga, Arnoud Arntz, Saskia van Es (?)
2. Participants’ views on central mechanisms of EMDR and ImRs, a qualitative study
   - First author: Simone Menninga
   - Co-authors: Katrina Boterhoven de Haan, Chris Lee, Arnoud Arntz (senior?), Saskia van Es
3. Change in Schema Modes along treatment
   - First author: Martine Daniels
   - Co-authors: Mariel Meewisse, Eva Fassbinder, Marisol Voncken
4. Essential ingredients of ImRs: an observational study
   - First author: Thera Koetsier
   - Co-authors: Mariel Meewisse, Arnoud Arntz, Marisol Voncken, Eva Fassbinder
5. Treatment integrity
   - First author: Chris Lee
   - Co-authors: Open
6. Change in specificity of memories
   - First author: Amina Memon
   - Co-authors: Chris Lee, Marisol Voncken, Eva Fassbinder, Arnoud Arntz
7. Validity of the EMDR Fidelity Checklist
   - First author: Chris Lee
   - Co-authors: Open
8. Qualitative Changes in Memory Reconsolidation Following Treatment
   - First author: Student
   - Co-authors: Chris Lee
9. Therapist’s views on treatment for PTSD related to childhood trauma
   - First author: Katrina Boterhoven de Haan
   - Co-authors: Chris Lee (senior), Helen Correia
10. Prediction of effects and dropout
    - First author: Eva
    - Co-authors: study board

Note: co-authors lists not necessarily complete, people can join substudy groups.

Appendix 2. World Health Organization Trial Registration Data Set

| **DATA CATEGORY** | **INFORMATION** |
| --- | --- |
| Primary registry and trial identifying number | ANZCTR.org.au ACTRN12614000750684 |
| Date of registration in primary registry | 16 July 2014 |
| Secondary identifying numbers |  |
| Source(s) of monetary or material support | EMDR Research Foundation |
| Primary sponsor | University of Western Australia |
| Secondary sponsor(s) | Sexual Assault Resource Centre |
| Contact for public queries | *AA, CL, EF* |
| Contact for scientific queries | *AA, CL, EF* |
| Public title | Treatment of Childhood Trauma: What Works Best in Reducing Trauma Symptoms for People Who Experienced a Traumatic Event in Childhood |
| Scientific title | International multi-centre RCT comparing the effectiveness of Imagery Rescripting and Eye Movement Desensitisation and Reprocessing as treatment for adults with Post-Traumatic Stress Disorder (PTSD) related to childhood trauma in reducing severity of PTSD symptoms and improving quality of life |
| Countries of recruitment | Australia, Germany and The Netherlands |
| Health condition(s) or problem(s) studied | Post-traumatic stress disorder |
| Intervention(s) | Active comparator: Imagery Rescripting (ImRs) and Eye-Movement Desensitisation and Reprocessing (EMDR) |
| Key inclusion and exclusion criteria | Ages eligible for study: 18 - 70 years Inclusion: DSM-IV diagnosis of PTSD, as assessed with the SCID-II or MINI PTSD as main complaint PTSD diagnosis lasting for 3 months or more Trauma that occurred prior to the age of 16 years old and participant agrees that this is the main focus of treatment If recent trauma - happened more than 6 months ago Aged between 18 and 70 years old Able to understand, read, and write country of participation language (Dutch, German or English)  Exclusion criteria: Acute PTSD DSM-IV diagnosed alcohol or drug dependence Benzodiazepine use (participant can participate if they stop benzodiazepine and they have been abstinent for at least 2 weeks) Comorbid psychotic disorder DSM-IV Bipolar disorder, type 1 Acute suicide risk IQ of 80 or less Scheduled to begin another form of PTSD treatment PTSD focused treatment within the past 3 months Participants should not start/continue any form of psychological therapy or medication during the screening, treatment, and waitlist stage (up to 8 weeks post-treatment assessment) Changes in medication within last 3 months Not able to plan 12 x 90 minutes treatment sessions twice a week within 6-8 weeks |
| Study type | Interventional Allocation: randomized Intervention model: parallel assignment Masking: blinding of research assistants  Primary purpose: prevention Phase N/A |
| Date of first enrolment | October 2014 |
| Target sample size | 142 |
| Recruitment status | Recruiting |
| Primary outcome(s) | Change in PTSD symptom severity shortly after the intervention phase (assessed at 8-week post treatment follow up), compared to severity of PTSD symptoms at the pre-treatment phase. |
| Key secondary outcomes | Change in depression, trauma-related guilt, trauma-related shame, anger, dissociation and functioning. |

Appendix 3. Consent form example

**FORM OF CONSENT**

1. I have read the Information Sheet provided explaining the study titled “Treatment of Childhood Trauma” and been given a full explanation of the purpose of this study, the procedures involved and of what is expected of me.
2. I agree voluntarily to take part in this study.
3. I understand that I will be asked to undergo psychological treatment using either Imagery Rescripting or Eye Movement Desensitization and Reprocessing. This will require me to:
   - Attend a maximum of 12, 90-minute sessions twice a week of either Imagery Rescripting or Eye Movement Desensitization and Reprocessing
   - Complete self-report questionnaires and be interviewed at different points including: prior to starting treatment, after treatment has finished, 8 weeks after treatment finished, and at a 1-year follow up.
4. I understand that I may be asked to attend an additional interview after my treatment has finished.
5. The researcher has answered all my questions and has explained possible problems that may arise as a result of my participation in this study.
6. I understand I am free to withdraw from the study at any time prior to the conclusion of the treatment, before the end of the 12 sessions, without needing to give any reason.
7. I understand I will not be identified in any publication arising out of this study.
8. I understand that my name and identity will be stored separately from the data. All data provided by me will be analysed anonymously using code numbers.
9. I understand that this data may be used for future research to help better our understanding of the treatment of PTSD and of trauma memories.
10. Any data that is used in future research will not be able to identify me specifically.

Name of participant: ________________________

(Participant’s full name)

Signature of Participant: ________________________ Date: …..../..…../…….

I confirm that I have provided the Information Letter concerning this study to the above participant; I have explained the study and have answered all questions asked of me.

Name of researcher: ________________________

(Investigator’s full name)

Signature of researcher: ________________________ Date: …..../..…../…….

Appendix 4. Additional items added to the Impact of Events Scale Revised (IES-R)

|  | | **With respect to __________________________** | | | | |
| --- | --- | --- | --- | --- | --- | --- |
|  | | Not at  All | A little  Bit | Moderately | Quite a  Bit | Extremely |
| 23. | I had feelings of guilt about it. | 0 | 1 | 2 | 3 | 4 |
| 24. | I felt ashamed about it. | 0 | 1 | 2 | 3 | 4 |
| 25. | I felt angry about it. | 0 | 1 | 2 | 3 | 4 |
| 26. | I felt disgust about it. | 0 | 1 | 2 | 3 | 4 |

Appendix 5. Additional items added to the Life Events Checklist (LEC-5)

**EXTRA ITEMS LIFE EVENTS CHECKLIST FOR THE DSM-5**

Be sure to consider your *entire life* (growing up as well as adulthood) as you go through the list of events.

|  | \| ***Event*** \| \| --- \| | \| ***Happened to me*** \| \| --- \| | \| ***Witnessed it*** \| \| --- \| | \| ***Learned about it*** \| \| --- \| | \| ***Part of my job*** \| \| --- \| | \| ***Not Sure*** \| \| --- \| | \| ***Doesn’t Apply*** \| \| --- \| |
| --- | --- | --- | --- | --- | --- | --- | --- | --- | --- | --- | --- | --- | --- | --- |
| **1.** | **Emotional abuse (like severely bullied, humiliated, yelled at, verbally threatened, punished in a unfair or cruel way)** |  |  |  |  |  |  |
| **2.** | **Emotional neglect (like taking care of parents or other children in the family, parents were addicted to alcohol or drugs, being left to your own devices)** |  |  |  |  |  |  |
| **3.** | **Physical neglect (like not getting enough food, or need to provide for your own food, left home alone during the day or at night under age of 12, not getting the medical care that was necessary)** |  |  |  |  |  |  |
